# Palatal asymmetry assessed by intraoral scans: effects of sex, orthodontic treatment, and twinning. A retrospective cohort study

**DOI:** 10.1186/s12903-023-02993-1

**Published:** 2023-05-18

**Authors:** Botond Simon, Francesco Guido Mangano, Adrienn Pál, István Simon, Dalma Pellei, Arvin Shahbazi, János Vág

**Affiliations:** 1 Department of Restorative Dentistry and Endodontics, Semmelweis University, Budapest, Hungary; 2 Department of Pediatric, Preventive Dentistry and Orthodontics, Sechenov First State Medical University, Moscow, Russia; 3 Department of Paediatric Dentistry and Orthodontics, Semmelweis University, Budapest, Hungary; 4 Department of Anatomy, Histology and Embryology (Oral Morphology Group), Semmelweis University, Budapest, Hungary; 5 Department of Periodontology, Semmelweis University, Budapest, Hungary; 6 Department of Prosthodontics, Semmelweis University, Budapest, Hungary

**Keywords:** Palate, Symmetry, Intraoral scanner, Twins, Heritability, Sex, Orthodontic treatment

## Abstract

**Background:**

Symmetry is critical in perceived attractiveness, especially in female faces. The palate determines the teeth’ alignment and supports facial soft tissues. Therefore, the study aimed to assess the effects of sex, orthodontic treatment, age, and heritability on the directional, anti-, and fluctuational asymmetry in the digital palatal model.

**Methods:**

The palate of 113 twins, 86 female and 27 male subjects, with and without previous orthodontic treatment, were scanned by the Emerald (Planmeca) intraoral scanner. Three lines were constructed horizontally in the digital model, one between the right and left first upper molars and two between the first molars and incisive papilla. Two observers calculated the left and right angles between the mid-sagittal plane and molar-papilla lines. The intraclass correlation coefficient was used to assess the inter-observer absolute agreement. The directional symmetry was determined by comparing the mean left and right angles. The antisymmetry was estimated from the distribution curve of the signed side difference. The fluctuating asymmetry was approximated from the magnitude of the absolute side difference. Finally, the genetic background was assessed by correlating the absolute side difference between monozygotic twin siblings.

**Results:**

The right angle (31.1 degrees) was not significantly different from the left one (31.6 degrees). The signed side difference followed a normal distribution with a mean of -0.48 degrees. The absolute side difference (2.29 degrees, p < 0.001) was significantly different from zero and negatively correlated (r=-0.46, p < 0.05) between siblings. None of the asymmetries was affected by sex, orthodontic treatment or age.

**Conclusions:**

The palate illustrates neither directional asymmetry nor antisymmetry, indicating that most people’s palates are symmetric. However, the significant fluctuating asymmetry suggests that some subject has considerable asymmetry but is not influenced by sex, orthodontic treatment, age, and genetics. The proposed digital method is a reliable and non-invasive tool that could facilitate achieving a more symmetrical structure during orthodontic and aesthetic rehabilitation.

**Trial registration:**

The Clinicatrial.gov registration number is NCT05349942 (27/04/2022).

## Background

Attraction or beauty is the appearance of external features [1]. The asymmetries or deformities are often driven due to developmental and genetic disorders [2]. Therefore, symmetry greatly influences the mate choice and the subjective judgment of fellow human beings [3]. Asymmetry close to the midline could significantly decrease aesthetic, while slight asymmetry in the lateral areas could be beneficial [4, 5]. Human beings show bilateral symmetry on the outside. However, in static 2D portraits, the mirrored faces differed by 31% on average from the original frontal plane [6]. More than 50% of healthy people have an asymmetry of at least 2 mm, and the differences occur primarily in the frontal (coronal) plane [7].

Reconstruction and improvement of symmetry are essential during orthognathic [8] and cleft surgeries [9]. 3D planning significantly improves the symmetry in these surgeries over 2D planning [10]. However, numerous cases still suffer facial asymmetry after bilateral surgery [11]. Furthermore, the residual asymmetry correlates with preoperative asymmetry [11], indicating the significance of evaluating skeletal asymmetry before the treatment. In addition, the underlining hard tissue partly determines the facial (soft tissue) symmetry [3, 8]. However, repositioning the maxilla by Le Fort I osteotomy does not change the internal asymmetry of the palate. Therefore, orthodontic treatment of underlining hard tissue such as the palate should establish symmetry by non-surgical orthodontic treatment of palatal expansion [12]. Nevertheless, the prevalence and methodology of palatal asymmetry are neglected in the literature [13].

Van Valen [14] categorized the deviation from symmetry into three types. i**) Directional asymmetry** occurs when a character is larger on one side than the other. (e.g., mammalian heart). Statistically, the mean value differs systematically between the two sides. (ii) **Antisymmetry** indicates that asymmetry is detected in most individuals, but the dominant side varies between individuals. It can be captured by bimodal distribution or platykurtosis [15]. (iii) **Fluctuating** asymmetry represents a random pattern. Statistically, the signed differences between the sides follow the normal distribution, with an equal mean size of the sides. Additionally, the three asymmetries can be combined in the same person. Directional asymmetry and antisymmetry are strictly hereditary [16], while the third type reflects the modulating effect of the environment and inaccuracy in development [17]. Due to the two X chromosomes of women, they are less prone to develop asymmetry than men. This phenomenon might be used in a forensic investigation for the sex determination of human remains. However, sex differences in dental asymmetry are contradictory [3].

Investigation of symmetry could be performed on 2D (e.g., X-ray, photographs) or 3D images (e.g., cone beam computed tomography, 3D surface laser scanning, 3D stereophotogrammetry). Nevertheless, the 2D and 3D evaluations could be discordant [18]. Although adjusting computer tomography could reduce radiation exposure in orthodontic treatment while still obtaining valuable measurements [19, 20], continuous symmetry monitoring during orthodontic treatment can not be done due to the radiation. However, scanning the complete arch, including the soft tissues by an intraoral scanner became highly reliable [21, 22]. Therefore it offers a non-invasive, easy opportunity to analyze the dimension of the mouth [23].

The study aimed to investigate the reliability of the asymmetry measurement on the digital palatal model. The second aim was to examine the effect of sex, orthodontic treatment and age on directional asymmetry, fluctuating asymmetry, and antisymmetry. Third, the heritability of the asymmetry was assessed by the correlation between monozygotic twin siblings.

## Methods

### Participants

One hundred seventy-four subjects were recruited from the National Twin Registry [24, 25]. Zygosity was determined by a questionnaire [26]. Accordingly, the participants comprised 61 monozygotic pairs (122 individuals) and 26 same-sex dizygotic twin pairs (52 individuals). The study was registered in ClinicalTrials.gov, registration number: NCT05349942 (27/04/2022). In addition, the evidence of previous orthodontic treatment was recorded. Each participant’s palate was scanned three times using a Planmeca Emerald intraoral scanner (Planmeca Oy, Helsinki, Finland, version number Romexis 5.2.1) as previously described [21].

The inclusion criteria were an intraoral scan without flaws, confirmed zygosity, and an age above 16. The exclusion criteria were maxillary expansion (including non-surgical and surgical), missing first molars on either side, triplets (having more than one sibling), Marfan syndrome, or any extremity of the palatal vault. Sixty-one subjects were excluded due to the missing landmark from the scan, or the previous orthodontic treatment could not be ascertained. The mean age of the included volunteers was 29 (17–65 years), with 86 females and 27 males (Table 1.)

**Table 1:** The age (years) distribution of groups

sex	orthodontic treatment	sample size	mean	SD	minimum	maximum
male	No	18	26	10	17	45
	Yes	9	31	9	18	45
female	No	37	34	16	17	65
	Yes	49	26	7	19	51

SD, standard deviation

### Angle measurement in the horizontal plane

**Fig. 1:**
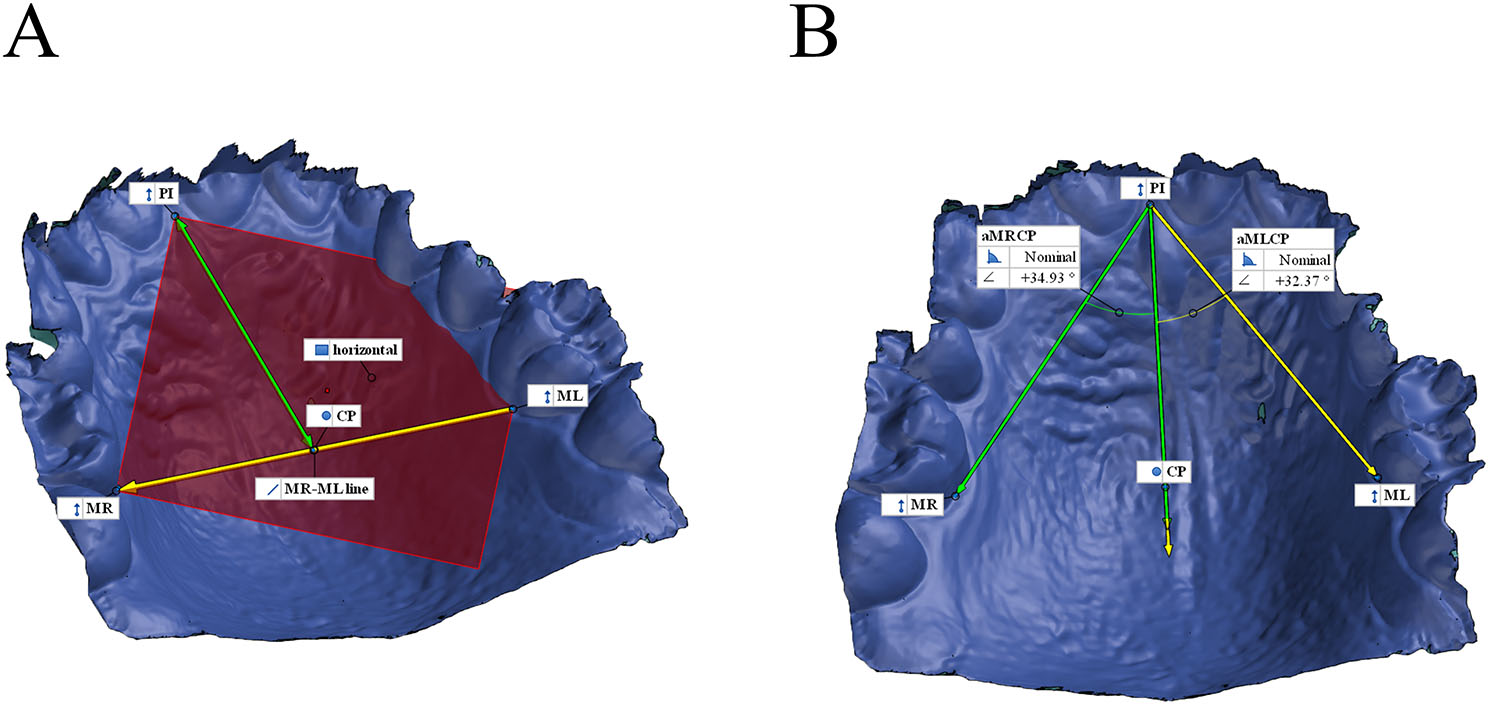
The construction of the landmark for the determination of asymmetry. (**A**) ML, left molar; MR, right molar; PI, the mesial tip of the papilla; CP, the center of the palate in the horizontal plane. The PI, MR, and ML created the horizontal plane (red). A line (green) perpendicular to the MR-ML line was projected to the PI. The intersection point of this mid-palatal line and the MR-ML line determined the CP. (**B**) The angle on the right side was defined between PI-CP and PI-MR line (aMRCP, green). The angle on the left side was defined between PI-CP and PI-ML line (aMLCP, yellow)

The angles were measured in one randomly selected scan replicate of 113 subjects in the GOM Inspect Suite (GOM GmbH, Braunschweig, Germany, software version 2020). Two observers performed the angle measurements. Each observer independently selected the following points, the anterior tip of the incisive papilla (PI), the intersection point of the extension of the palatal groove on the first molars to the gingival margin, and the line of the marginal gingiva (ML, MR). The three points defined the horizontal plane (Fig. 1). A line (green) laying on the horizontal plane perpendicular to the MR-ML line was projected to the PI. The intersection point of this mid-palatal line and the MR-ML line determined the center of the palate (CP). The relation of ML-PI-CP and MR-PI-CP determined the left and right angles (aMRCP, aMLCP). The angle measurement is independent of the palatal size, contrary to the distance measurement. Therefore, it allows the standard comparison of the larger and smaller palate (e.g., comparison of male and female) [18].

### Reliability of the angle measurement

The angles followed the normal distribution. Therefore, they were given in the text as mean ± standard error (SE). A two-way random-effects model was used to calculate the intraclass correlation coefficient (the absolute agreement) of angles between observers for the single (ICC(2,1)) and average observer (ICC(2,2)) [27]. The closer the intraclass correlation coefficient (ICC) is to 1, the more accurate the measurement. The ICC is a relative value with no unit of measurement. It expresses the accuracy relative to a given measurement range. The ICC (2,1) indicates what would happen if only one observer were measured. The ICC (2,2) shows what would happen if the measurement of multiple observers averaged. The standard error of the mean (typical error, SEM) was calculated according to Weir et al. [28]. The SEM estimates how repeated measures are distributed around the true score.

### Homogeneity of the groups and assessment of the directional asymmetry

Subjects were divided into four groups based on sex (female and male) and orthodontic treatment (orthodontic treated and non-treated). The mean angles (including left and right) between groups were compared by linear mixed model. The fixed factors were the ‘side’, ‘sex’, and ‘orthodontic treatment’. The two-way and three-way interaction terms were also included. The significance of the side (main) effect would indicate antisymmetry, i.e., one side is systematically more prominent than the other. The significance of the sex (main) effect would mean larger angles in one of the sex groups. The significance of the orthodontic (main) effect would indicate treatment affects the angles on both sides or the treated and non-treated groups were not homogenous. Significant interaction terms of ‘side*sex’ or ‘side*orthodontic treatment’ would indicate that directional asymmetry (i.e., differences between sides) varies between sexes or due to the orthodontic treatment.

### Assessment of the antisymmetry

The signed side difference was calculated by subtracting the angle on the left side from the angle on the right side. Then the distribution of signed values was analyzed by curve inspection and the Shapiro-Wilk test. The significant deviation from the normal distribution and recognition of a bimodal distribution would suggest antisymmetry.

### Assessment of the fluctuating asymmetry

The fluctuating asymmetry was assessed by calculating the absolute side difference for each subject. The generalized linear mixed model, with gamma distribution and log-link function, was used to test the effect of sexes and orthodontic treatment on the fluctuating asymmetry. The significant intercept of the model would indicate that the absolute side difference deviates from zero as a marker for fluctuating asymmetry.

The effect of age on the absolute side difference was evaluated by Spearman’s Rho correlation. The genetic effect on the fluctuating symmetry was assessed by Spearman’s Rho correlation between the two siblings of monozygotic pairs.

### Sample size estimation

In a previous study [29], the slightest discrepancy between the left and right sides of the face, which can be perceived by visual inspection (i.e., the eyelid position at rest) was 2 mm. The depth of the palate (the distance between PI and CP) was 28.6 mm, and the width (the length between ML and MR) was 34.8 mm [23]. The measurements were done in the same population as the current one. Accordingly, a 2 mm deviation corresponds to 4 degrees angle difference between the palatal sides calculated by the tangent function. The necessary sample size to detect 4 degrees differences in the absolute side difference was five at 0.05 alpha and 0.95 beta levels (Gpower software, version 3.1.9.6., Kiel University, Germany). The male with orthodontic treatment had the lowest sample number (n = 9).

All statistical analyses were made in IBM SPSS Statistics, Version 27 (Armonk, NY: IBM Corp., USA). A p-value less than 0.05 was considered statistically significant.

## Results

### Reliability of the measurement

The inter-observer error in manual angle measurement in the GOM Inspect software is shown in Table 1. The precision of the measurement improved by only 0.04 ICC if the two measurements were averaged. The SEM of the measured angle was less than 1 degree (Table 2.). The coefficient of variation was between 2.6 and 2.9% for the single observer and between 1.9 and 2.1% for the average observer. However, a slight but significant bias was observed. One observer measured the right side as 0.28 degrees smaller and the left side as 0.39 degrees larger.

**Table 2:** The inter-observer agreement for the angles (degree)

	Absolute agreement (ICC)		SEM (degree)		difference between observers (mm)			
	a single observer (ICC 2,1)	average observer (ICC 2,2)	single observer	average observer	mean	SE	p	
Right side	0.910	0.953	0.90	0.65	-0.28	0.12	0.023	
Left side	0.918	0.957	0.80	0.58	0.39	0.10	0.000	

ICC, intraclass correlation coefficient

SEM, standard error of measurement

SE, standard error of the mean

### Homogeneity of the groups and assessment of the directional asymmetry

The angles on the left and right sides of the four groups are shown in Table 3. No differences in the mean angle were observed between females and males (p = 0.774) and between orthodontic treated and non-treated groups (p = 0.491) (Table 3.). Therefore, the angles were similar in female and male groups and orthodontic treated and non-treated groups indicating homogeneity of the four groups.

**Table 3:** The angles (degree) of the left and right side

Orthodontic treatment	sex		right angle	left angle	signed difference	absolute difference
No	Male (n = 18)	mean	31.7	31.2	0.50	2.21*
		SE	0.71	0.68	0.69	0.46
		SD	3.02	2.89	2.94	1.94
	Female (n = 37)	mean	31.7	31.9	-0.26	2.17*
		SE	0.54	0.54	0.46	0.29
		SD	3.26	3.27	2.80	1.75
Yes	Male (n = 9)	mean	30.3	32.3	-1.99	2.76*
		SE	0.85	0.73	0.73	0.29
		SD	2.55	2.19	2.20	0.87
	Female (n = 49)	mean	30.7	30.8	-0.15	2.02*
		SE	0.42	0.38	0.38	0.24
		SD	2.95	2.67	2.65	1.69

SE, standard error of the mean

SD, standard deviation

* indicates a significant difference from zero, p < 0.001

**Fig. 2:**
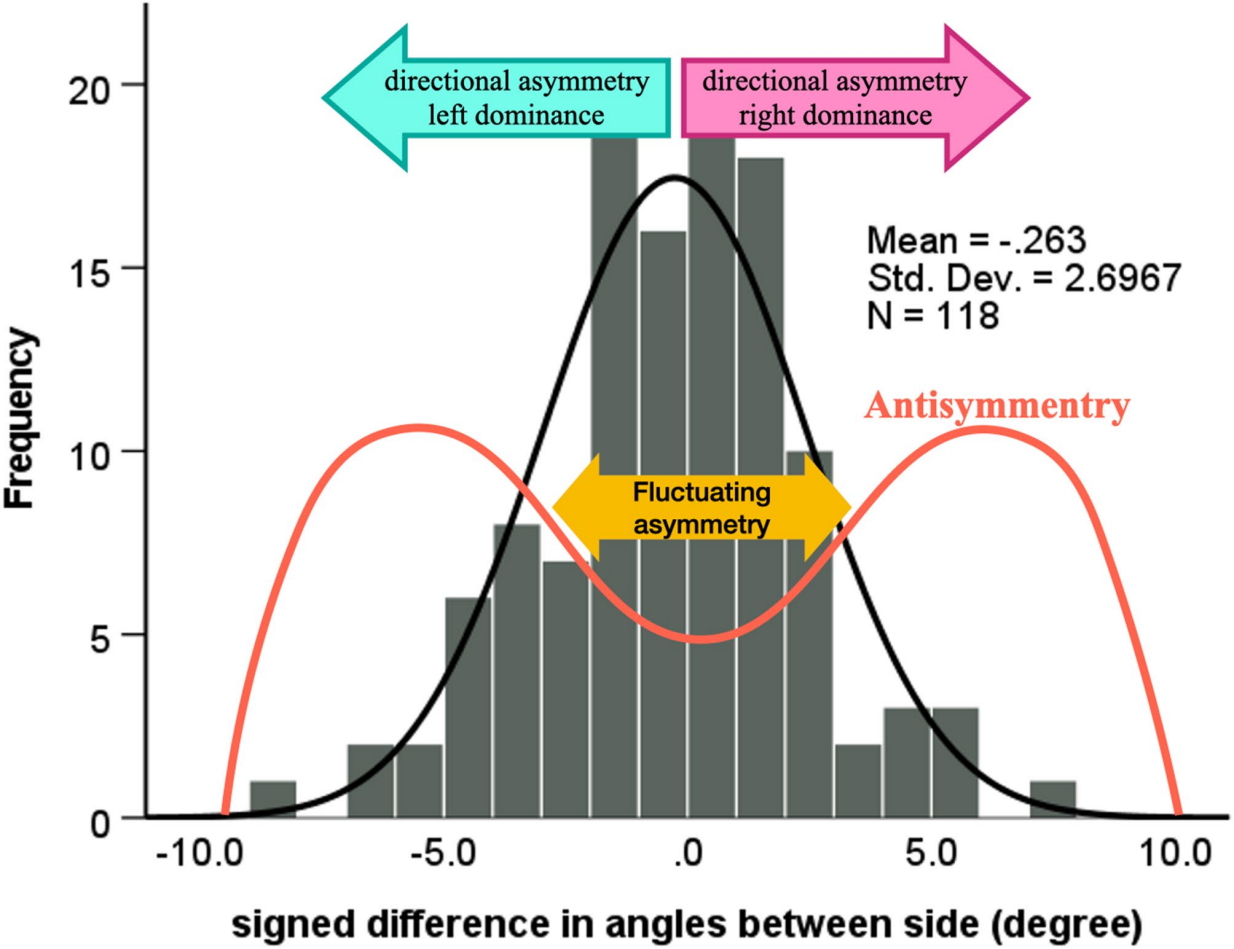
The measured signed difference values between the left and right angles (bars) follow the normal distribution (black curve) suggesting a lack of antisymmetry. A theoretical antisymmetry distribution would follow a bimodal distribution (orange line). The mean (the peak of the black curve) signed difference did not deviate from zero, indicating any directional asymmetry. However, a significant population had a considerable deviation from zero indicating fluctuating asymmetry

The left and right angles did not differ significantly (p = 0.221), indicating a lack of directional asymmetry. In addition, no significant two-way or three-way interaction was found between side, sex, and orthodontic treatment (Table 4.), designating no effect of sex and orthodontic treatment (Fig. 2.).

**Table 4:** The fixed effects of linear mixed model

Source	F	df1	df2	Sig.(p=)
side	1.499	1	920	0.221
sex	0.083	1	920	0.774
orthodontic treatment	0.711	2	920	0.491
side * sex	0.141	1	920	0.707
side * orthodontic treatment	1.889	2	920	0.152
sex * orthodontic treatment	0.415	2	920	0.660
side * sex * orthodontic treatment	2.137	2	920	0.119

### Assessment of antisymmetry

The distribution of the signed side difference did not deviate from the normal one (p = 0.597) (Fig. 2.). The skewness was negligible (-0.087 with a SE of 0.223), and the kurtosis was slightly positive (0.519 with a SE of 0.442). No bimodal distribution or platykurtosis was observed, indicating the absence of antisymmetry.

### Assessment of fluctuating asymmetry

The intercept of the linear mixed model was statistically significant (0.58 degrees, p < 0.001), indicating the existence of fluctuating asymmetry. 14% of the people have a higher deviation in either direction than the 4.0 degrees. The variation ranged between − 8.2 and 7.2 degrees.

However, neither the sex (p = 0.838) nor the orthodontic treatment (p = 0.682) main effect was significant. Furthermore, no interaction was found between sex and orthodontic treatment (p = 0.190). Thus, the fluctuating asymmetry is similar between sex and orthodontic groups.

No significant correlation was found (r = 0.29, p = 0.100) between age and the absolute side difference.

### Heritability of fluctuating asymmetry

A negative (moderate) correlation in absolute side difference (n = 19, r=-0.46, p < 0.05) between sibling A and sibling B in a non-orthodontic group of monozygotic twins was found. It means that the more symmetric sibling A is, the less symmetric sibling B is.

## Discussion

Digital dentistry recreated an integral position in analyzing palatal symmetry. According to the results, the angle could be measured with high precision (less than one-degree error) on a 3D digital palatal model. The inter-observer error was substantially lower than the measurement error on the face [30]. Therefore, the 3D palatal model measurement could be a reliable method to evaluate anthropology, symmetry, and the effect of orthodontic treatment. The male has a larger palate than females [23, 31], but no difference was found in the angles between females and males. Therefore, the angle measurement could successfully eliminate the inequality in palate size. Consequently, it could be used to measure symmetry in different populations.

No directional asymmetry was encountered since the mean angle was not different between the left and right sides, suggesting no dominant palatal side in any investigated groups (female, male, orthodontic treatment, not-treated). Antisymmetry was also rejected since the distribution of the signed difference did not show bimodal distribution or platykurtosis. Therefore, contrary to handedness, most people cannot be categorized into the left or right palatal dominant group. However, the digital palatal model analysis revealed the fluctuating asymmetry. 14% of people have more than 4 degrees of deviation on one side corresponding to 2 mm mean deviation (visual perception limit) [29]. The maximum difference was more than 8 degrees, corresponding to 4 mm in width as calculated from the population mean palate depth, given previously [23], and the tangent function.

**Sex** did not influence fluctuating symmetry, similar to a previous study on the face [32]. It was unexpected that the **orthodontic treatment** did not affect symmetry either since orthodontic treatment supposes to improve esthetics. A possible explanation could be that facial asymmetry can be attenuated by a camouflaging orthodontic treatment. The treatment can satisfy the patient without correcting the jaws [5, 33]. Presumably, the symmetry of the palate does not improve during orthodontic treatment without palatal expansion. The detailed orthodontic treatment (time, multiband type, number of misaligned teeth, malocclusion) was not evaluated. However, our study did not include severe orthodontic discrepancy requiring orthognathic [8] and cleft surgeries [9]. The rapid maxillary expansion increased the palatal dimension sagitally and transversally significantly, but the difference between sides and the effect of it on symmetry was not evaluated [20, 34]. Low-dose CT pre- and postoperative facilitates diagnosis [19]; thus, it might be used for diagnosis and symmetry assessment. However, the current study proposed a non-invasive and reliable method to aid in adjusting the treatment and achieving predictable and sustainable facial symmetry. For this purpose, an intraoral scan incorporating the palate can easily and quickly be analyzed without repeated radiation.

In a recent study [13], treating the unilateral cross-bite with a bio-activator (AMCOP, Ortho Protec, BA, Italy) significantly improve the symmetry of the palate. The differences in intermolar widths between the cross-bite and non-crossbite sides decreased from 1.54 to 0.088 mm. The pre-treatment values (1.54 mm) are somewhat smaller than the mean absolute side difference in the current study (2.48 mm), but Lo Giudice et al. investigated children with a mean age of 7. Notable, the maxillary arch constantly changes with age [35, 36]. In the current investigations, 18 patients had higher than 4 mm differences regardless of orthodontic treatment. However, the asymmetry (mandibular shift > 2 mm) was an inclusion criterion in Lo Guidice et al.’s study, whereas, in our retrospective study, the orthodontist was unaware of the palatal or any craniofacial asymmetry. Consequently, it is suggested to measure the asymmetry before the treatment to incorporate its correction in the treatment plan [37]. Another conclusion of these results is that cross-bite might be an etiology factor in palatal asymmetry [37]. However, habitual mastication on one side might be another possible factor. Furthermore, a bad habit could relapse the results of the orthodontic treatment; therefore, it is essential to recognize it before the treatment.

Age did not affect the symmetry either. However, decreased maxillary arch length was observed after the age of 13 previously [35, 36], but it might occur similarly on the two sides.

Our results also show that when one sibling is less symmetric, the other is more symmetric, suggesting that asymmetry (or symmetry) may be a complementary trait. [38]. The inverse correlation suggests the lack of any genetic effect in the expression of symmetry. The environmental factors, the mutations that occur during development [17], and the individual’s response to these influences [39, 40] might determine the degree of symmetry. Genetic and environmental factors influence the umbilical cord development; thus, siblings might be subject to different influences from a very early stage of life [41]. In twin-twin transfusion syndrome, which occurs in 15% of monochorionic monozygotic twins, one sibling receives a better blood supply than the other [42]. Consequently, one twin could be larger than the other.

## Conclusion

Digital models obtained with an intraoral scanner can be a reliable tool for evaluating the symmetry of the palate. There is no dominant side of the palate in the Caucasian population. Most people have no dominant side either. Therefore they are relatively symmetric. However, a small percentage of people have some perceptible asymmetry in the palate, which is not influenced by conservative orthodontic treatment. The lack of effect of sex on symmetry and the inverse correlation in twin siblings suggest that the expression of symmetry is an environmentally driven phenomenon.

## Data Availability

The datasets used and/or analysed during the current study available from the corresponding author on reasonable request.

## List of Abbreviations

CP: Center of the palate
ICC: Intraclass correlation coefficient
ML: Molar left
MR: Molar right
PI: Incisive papilla
SD: Standard deviation
SE: Standard error of the mean
SEM: Standard error of the measurement
